# 4-Octyl itaconate inhibits poly(I:C)-induced interferon-β secretion in mouse bone marrow-derived macrophages partially by activating Nrf2

**DOI:** 10.1016/j.heliyon.2023.e23001

**Published:** 2023-12-01

**Authors:** Ying-Xing Yue, Bing-Bing Jia, Ji-Rong Wang, Ying-Zheng Weng, Gen-Xiang Mao, Yan Lu, Jing Yan, Zhou-Xin Yang

**Affiliations:** Zhejiang Provincial Key Lab of Geriatrics and Geriatrics Institute of Zhejiang Province, Department of Geriatrics, Zhejiang Hospital, 1229 Gudun Road, Hangzhou, China

**Keywords:** 4-Octoyl itaconate, Interferon-β, Macrophages, Nrf2

## Abstract

Viruses have become a major threat to human health. Interferon-β (IFN-β) has a key role in the antivirus process, as it can increase the expression of antivirus-associated genes. Itaconate and its derivatives can regulate the immune response, secretion of inflammatory factors, and pyroptosis of macrophages. The effect of itaconate on IFN-β secretion of double-stranded RNA-induced macrophages are not well known. A derivative of itaconate, 4-octoyl itaconate (4-OI), was used to treat mouse bone marrow-derived macrophages (BMDM) induced with 100 μg/mL poly(I:C). The IFN-β concentration was detected through ELISA, and IFN-β mRNA expression was detected through quantitative PCR. High-throughput transcriptome sequencing was used to analyze changes in the BMDM transcriptome after 4-OI treatment. The Nrf2 expression was knocked down with siRNA.4-OI inhibited poly(I:C)-induced IFN-β secretion and mRNA expression in BMDM. Results of transcriptome sequencing revealed that 4-OI downregulated 1047 genes and upregulated 822 genes. GO and KEGG enrichment of differently expressed genes revealed that many downregulated genes were related to the anti-virus process, whereas many upregulated genes were related to metabolism. The Nrf2 inhibitor ML385 and Nrf2 siRNA could partially reverse the inhibitory effect of 4-OI. In conclusion, 4-octyl itaconate could inhibit the poly(I:C)-induced interferon-β secretion in BMDM partially by regulating Nrf2.

## 1 Introduction

In recent years, SARS-COV-2, H1N1, and other viruses have spread widely around the world, greatly increasing mortality rates globally. Viruses have become one of the major threats to human health [1,2]. After the virus infects the host, the innate immune response of the host to the virus is a critical defense response in the early stages of infection. Among the responses induced, type I interferons such as IFN-β are induced after viral infection [3] and play a key role in the antivirus process, as they can increase the expression of antivirus-associated genes [4,5].

Innate immune cells such as macrophages are important antivirus cells and have a crucial role in the antivirus immune response [6]. Macrophages can secrete IFN-β after devouring virus-infected cells or virus particles [7], thereby activating the response to the virus and inhibiting virus proliferation in vivo. TLR3 recognizes double-stranded RNA or the RNA complex structure of viruses, which is a vital process to fight against RNA viruses, and its downstream activates inflammatory factors and IFN-β [8]. Poly(I:C) is often used as a mimic of double-stranded RNA and to stimulate the IFN-β production by macrophages [9]. Although the key role of IFN-β in the antiviral response has been well recognized, IFN-β regulation remains undefined.

Itaconate is a crucial metabolite, and its content is mainly determined on the basis of the aconitate decarboxylase 1 (ACOD1) levels [10]. After infection, the ACOD1 expression increases rapidly, and, aconitate, which is a major product in the tricarboxylic acid (TCA) cycle, is transformed into itaconate [10]. Itaconate and its derivatives such as dimethyl itaconate (DI) and 4-octyl itaconate (4-OI) can regulate the immune response, the secretion of inflammatory factors, and pyroptosis of macrophages [[11], [12], [13]]. 4-OI could regulate lipopolysaccharide-induced IFN-β secretion [14].

In this study, we explored the effect of 4-OI on IFN-β secretion of poly(I:C)-induced bone marrow-derived macrophages (BMDM), analyzed the overall changes triggered by 4-OI in the gene expression of these BMDM through transcriptome sequencing and investigated the role of Nrf2.

## 2 Methods

### 2.1 Mouse bone marrow-derived macrophages (BMDM) culture

Male C57/BL6 mice (aged 6–8 weeks) were purchased from the animal experiment center of the Hangzhou Medical College. The mice were executed and immersed in 75 % alcohol for 1 min. Then, the femurs were separated and both ends were cut off and rinsed with DMEM medium. After centrifugation at 1500 rpm for 5 min, the cells were resuspended in the DMEM medium (Thermo Fisher Scientific Inc., Waltham, MA USA) supplemented with 50 ng/mL M-CSF (R&D, Minneapolis, MN USA), 10 % FBS (Thermo Fisher Scientific Inc.) and 100 U/mL of penicillin/streptomycin, seeded into a 6-cm dish, and incubated at 37 °C (Thermo Fisher Scientific Inc.) containing 5 % CO_2_. After 2 days of culture, the cell suspension was removed and replaced with a fresh medium for further culture. Next, the medium was replaced every 2 days. After 7 days of culture, the adherent cells were repeatedly blown to form cell suspension, and the cell concentration was adjusted to 10^6^/mL for subsequent analyses. Then, 100 μg/mL poly(I:C) (Sigma-Aldrich, Inc., St. Louis, MO USA) was added to the culture medium to induce IFN-β. The ethics of the animal procedures were proved by the Institutional Animal Care and Use Committee, Zhejiang Center of Laboratory Animals (ZJCLA-IACUC-20140002).

### 2.2 Cell viability test

BMDM (5 × 10^3^/100 μL) was added to a well of 96-well plates and allowed to adhere for 24 h. Then, 0.03125, 0.0625, 0.125, or 0.25 mM 4-OI or vehicle control (Dimethyl sulfoxide, DMSO) was added to the wells for 24 h. After incubation for 2 h with the addition of 10 μL of cell counting kit-8 reagent (CCK-8, 7Sea Pharmatech Co. Ltd., Shanghai, China), the absorbance was measured at 450 nm by using a microplate reader.

### 2.3 Enzyme-linked immunosorbent assay (ELISA)

Cells (1 × 10^6^) were seeded into a well of a 6-well plate and incubated for 24 h. Specific treatments were given to each group. The cell supernatants were collected and centrifuged at 10000 rpm for 1 min. The supernatants were stored in a −80 °C refrigerator. The ELISA Kit for IFN-β was purchased from R&D, and the experiment was performed according to the kit instructions.

### 2.4 Quantitative polymerase chain reaction (PCR)

RNA extraction was performed by using the RNA-Quick Purification Kit (Yishan Biotechnology Co. Ltd., Shanghai, China). Reverse transcription was performed by using the ReverTra Ace qPCR RT Kit (Toyobo Inc., Osaka, Japan). Quantitative PCR was performed by using SYBR Green Real-time PCR Master Mix (Toyobo Inc.). Glyceraldehyde-3-phosphate dehydrogenase (Gapdh) was used as a control. The primers used in the study are given in Table 1.

**Table 1:** Primer sequences used in the study.

Target	Sequences
*Ifnb* Forward	5′-CAGCCCTCTCCATCAACTATAAG-3′
*Ifnb* Reverse	5′-CCTGTAGGTGAGGTTGATCTTTC-3′
*Tnfsf15* Forward	5′-GATCATAGAAGCCCACGAGTTC-3′
*Tnfsf15* Reverse	5′-CATGGGACCGTGATTGAGTAAG-3′
*Ccl7* Forward	5′-CCAGCTCTCTCACTCTCTTTCT-3′
*Ccl7* Reverse	5′-CCCACACTTGGATGCTGAAA-3′
*Gm6377* Forward	5′-GAAGCCCTTCTCCACTCTTTAG-3′
*Gm6377* Reverse	5′-TTCCCGACTAGCTTGGTTTG-3′
*Klf7* Forward	5′-GAGCAGTTAAGAGTGGACAGAG-3′
*Klf7* Reverse	5′-TTTCCGGCACCCGTTAAA-3′
*Edn1* Forwards	5′-TCTCTCTGCTGTTTGTGGCT-3′
*Edn1* Reverse	5′-CCAGGTGGCAGAAGTAGACA-3′
*Cdkn2b* Forward	5′-TCCGCAAGGACTTCTTTCTC-3′
*Cdkn2b* Reverse	5′-GTCTTACTGGGTAGGGTTCAAG-3′
*Hmox1* Forward	5′-CTCTCTTCTCTTGGGCCTCTAA-3′
*Hmox1* Reverse	5′-TGTCAGGTATCTCCCTCCATTC-3′
*Ypel5* Forward	5′-GGTCTGTGCTTGGGTCATTAT-3′
*Ypel5* Reverse	5′-AGTCAAATCTGGCCTCTTTCC-3′
*Gss* forwards	5′-AGACCAAAGAAGCTTCCAAGAT-3′
*Gss* reverse	5′-ACCGCATTAGCTGAGCCATA-3′
*Layn* forwards	5′-CACATCACAGTTTAGGAACTGG-3′
*Layn* reverse	5′-GATGGCTGATGGTACATGAC-3′
*Nrf2* Forward	5′-TTTTCCATTCCCGAATTACAGT-3′
*Nrf2* Reverse	5′-AGGAGATCGATGAGTAAAAATGGT-3′
*Gapdh* forwards	5′-ATCAACGACCCCTTCATTGACC-3′
*Gapdh* reverse	5′-CCAGTAGACTCCACGACATACTCAGC-3′

### 2.5 Transcriptome sequencing and GO/KEGG enrichment

BMDM from 3 mice was used for the treatment and transcriptome sequencing was performed by Metware Biotechnology Co., Ltd. (Wuhan, Hubei, China). Total RNA was extracted by using the Trizol kit. Sequencing libraries were generated by the NEBNext® UltraTM RNA Library Prep Kit for Illumina and were sequenced on an Illumina platform (Illumina, Inc., San Diego, CA, USA). FeatureCounts v1.6.2/StringTie v1.3.4d were used to calculate the gene alignment and fragments per kilobase million (FPKM). Genes with the corrected P < 0.05 and |log_2_fold-change|≥1 were recognized as differentially expressed genes using DESeq2 v1.22.1/edgeR v3.24.3.GO and KEGG enrichment was performed based on the hypergeometric test.

### 2.6 Western blotting

Macrophages were washed with PBS, transferred to a 1.5-mL centrifuge tube, added to the cell lysate, mixed uniformly, and placed on an ice bath for 30 min. The cells were centrifuged at 12000 rpm for 20 min at 4 °C and the supernatant was collected. The protein in the supernatant was concentrated and detected and then subjected to protein concentration determination by using the BCA kit (Beyotime Biotechnology, Shanghai, China). Finally, a sample loading buffer was prepared, the proteins were separated by SDS-PAGE electrophoresis and wet transfer to a polyvinylidene fluoride (PDVF) membrane (Merck Millipore, Darmstadt, Germany). After blocking with 5 % BSA, they were washed in PBST. The membranes were then incubated with primary antibody at room temperature at 4 °C overnight. Primary antibodies against Nrf2 (1:1000, Cell Signaling Technology, Massachusetts, USA, #12721) and GAPDH (1:5000， Cell Signaling Technology, #5174) were used for immunoblotting. The membranes were washed with PBST and incubated with HRP-linked anti-rabbit secondary antibody (1:5000, Cell Signaling Technology, #7074) at room temperature for 1 h. The membranes were washed with PBST and the expression of proteins was detected by using the ChemiDoc XRS Imaging System (Bio-Rad, California, USA). The relative gray value was calculated by the Image J software (National Institutes of Health, Maryland, USA).

### 2.7 siRNA interference

The cells were seeded into 6-well plates at the density of 1 × 10^6^ cells/well for 24 h for adherence. Before transfection, the media was changed with opti-MEM. Lipofectamine RNAiMAX Transfection Reagent (Thermo Scientific, USA) was used for transfection. Negative control (NC) and Nrf2 siRNA were purchased from Tsingke Biotechnology Co., Ltd. (Beijing, China). The final siRNA used per well was 100 pmol, and the final transfection reagent used per well was 9 μL. After 48 h of transfection, specific treatments were given to each group of BMDM.

### 2.8 Statistical analyses

The data were analyzed using GraphPad Prism 8.0 (Boston, MA USA) and presented as the mean ± standard deviation. The mean values between the two groups were compared by unpaired *t*-test. Analysis of variance was applied for comparison among ≥3 groups, and multiple tests of mean were performed using Tukey's test. p < 0.05 was considered to indicate a statistically significant difference.

## 3 Results

### 3.1 Effects of different 4-OI concentrations on BMDM viability

**Fig. 1:**
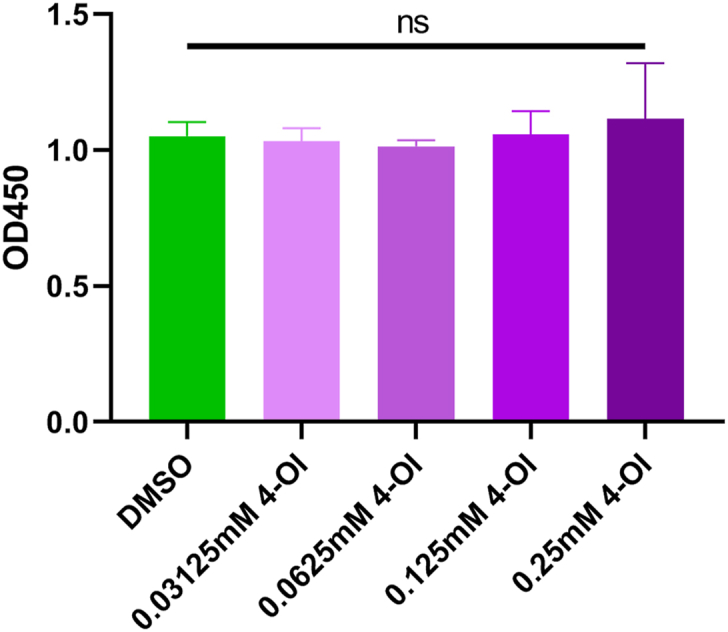
**Effect of 4-OI on cell viability of BMDM.** 0.03125, 0.0625, 0.125, and 0.25 mM 4-OI were added to the cultured BMDM for 24 h. CCK-8 kit was used to detect cell viability. Analysis of variance was applied, and multiple tests of mean were performed using Tukey's test. When compared to the DMSO group, ns, not significant, (n = 5).

The effects of different 4-OI concentrations on macrophage viability were compared. We examined BMDM viability after 24 h of treatment with 4-OI at 0.03125, 0.0625, 0.125, and 0.25 mM. No significant changes in OD values were detected after 4-OI addition (Fig. 1). These results suggested that 4-OI concentrations did not change BMDM viability.

### 3.2 4-OI inhibited poly(I: C)-induced IFN-β secretion and mRNA expression in BMDM

**Fig. 2:**
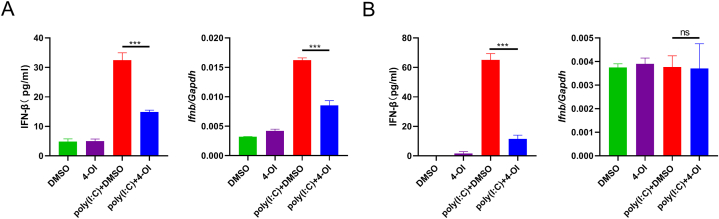
**4-OI regulates IFN-β secretion and mRNA expression in BMDM.** BMDM was pretreated with 0.25 mM of 4-OI or DMSO for 2 h, followed by the addition of poly (I:C) (final concentration 100 μg/mL) for 4 h (A) or 24 h (B). The concentration of IFN-β in the culture supernatant was detected by ELISA. The cells were collected, mRNA was extracted and reverted to cDNA, and the expression of IFN-β was detected by quantitative PCR. Analysis of variance was applied, and multiple tests of mean were performed using Tukey's test. ns, not significant, ***p < 0.001, (n = 3).

As 0.25 mM had no obvious effect on cell activity, we pretreated BMDM with 0.25 mM 4-OI for 2 h, followed by poly(I: C) induction for 4 h. ELISA showed that IFN-β secretion could be inhibited by 4-OI (p < 0.001). The quantitative PCR results showed that IFN-β mRNA expression was also significantly decreased (p < 0.001) (Fig. 2A). At 24 h after poly(I:C) induction, the IFN-β concentration in the supernatant of the 4-OI group was significantly different from that in the DMSO group (p < 0.001). However, the quantitative PCR results demonstrated that the effect of poly(I:C) on IFN-β expression was not obvious after 24 h, and 4-OI showed no obvious effect (p > 0.05) (Fig. 2B).

### 3.3 4-OI regulates the transcriptome of poly(I:C)-induced BMDM

**Fig. 3:**
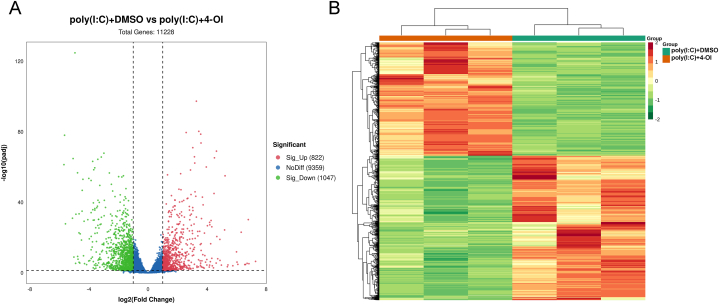
**4-OI regulated poly(I:C) induced changes in the transcriptome of macrophages.** BMDM in the culture state was pretreated with DMSO or 0.25 mM of 4-OI for 2 h, followed by the addition of poly (I:C) (final concentration 100 μg/mL) for 4 h, and the cells were collected for transcriptome analysis. A. Volcanic plots of the transcriptome-sequencing results. B. Transcriptome sequencing cluster analysis.

**Fig. 4:**
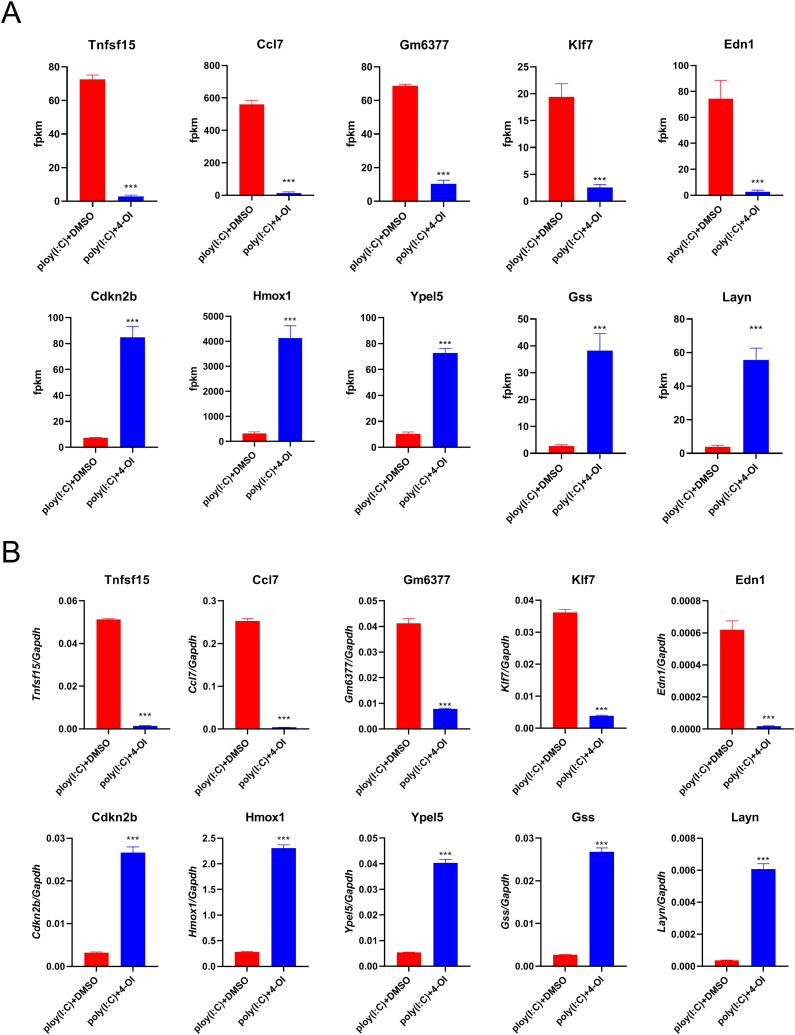
**Transcriptome sequencing results of TOP5 differential genes and quantitative PCR validation.** BMDM in the culture state were pretreated with DMSO or 0.25 mM of 4-OI for 2 h, followed by the addition of poly (I:C) (final concentration 100 μg/mL) for 4 h, and the cells were collected for transcriptome analysis and quantitative PCR. A. The fpkm values of genes with the greatest difference in Top5. B. Quantitative PCR was used to verify the expression level of the differential genes. The mean values between the two groups were compared by unpaired *t*-test. ***p < 0.001, (n = 3).

After 2 h of 4-OI treatment, IFN-β exhibited a large difference at the transcriptional level at this time point of 4 h of treatment with poly(I:C). Therefore, we used the cells at this time point for transcriptome analysis. In total, 1869 differential genes were identified. Of them, 1047 genes were downregulated and 822 genes were upregulated (Fig. 3A and B). According to the P value, the top 5 genes exhibiting the most significant downregulation were Tnfsf15, Ccl7, Gm6377, Klf7, and Edn1, whereas the top 5 genes exhibiting the most significant upregulation were Cdkn2b, Hmox1, Ypel5, Gss, and Layn (Fig. 4A). The expression of these 10 genes was verified through quantitative PCR (Fig. 4B, p < 0.001).

### 3.4 GO and KEGG enrichment of differently expressed genes for 4-OI-treated poly(I:C)-induced BMDM

**Fig. 5:**
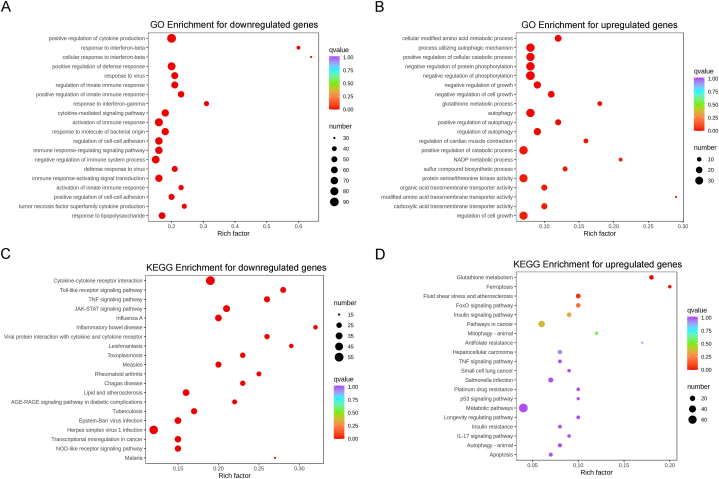
**GO and KEGG enrichment analysis results. A & B.** GO enrichment results for downregulated genes and upregulated genes. **C & D.** KEGG enrichment results for downregulated genes and upregulated genes.

GO enrichment was performed to explore the transcriptome results of poly(I:C)-induced BMDM treated with 4-OI. For downregulated genes, the top 5 GO-enriched terms with the lowest adjusted p value were positive regulation of cytokine production, response to IFN-β, cellular response to IFN-β, positive regulation of defense response, and response to the virus (Fig. 5A). For upregulated genes, the top 5 GO-enriched terms with the lowest adjusted p value were the cellular-modified amino acid metabolic process, process utilizing autophagic mechanism, positive regulation of the cellular catabolic process, negative regulation of protein phosphorylation and negative regulation of phosphorylation (Fig. 5B).

KEGG enrichment was also used to explore pathway-related changes occurring after 4-OI treatment. For downregulated genes, the top 5 KEGG-enriched pathways with the lowest adjusted P value were the cytokine–cytokine receptor interaction, toll-like receptor signaling pathway, TNF signaling pathway, JAK-STAT signaling pathway, and Influenza A (Fig. 5C). For upregulated genes, the top 5 KEGG-enriched pathways with the lowest adjusted P value were glutathione metabolism, ferroptosis, fluid shear stress and atherosclerosis, FoxO signaling pathway, and insulin signaling pathway (Fig. 5D).

### 3.5 Nrf2 inhibitor ML385 partially reversed the inhibitory effect of 4-OI on poly(I:C)-induced IFN-β secretion from BMDM

**Fig. 6:**
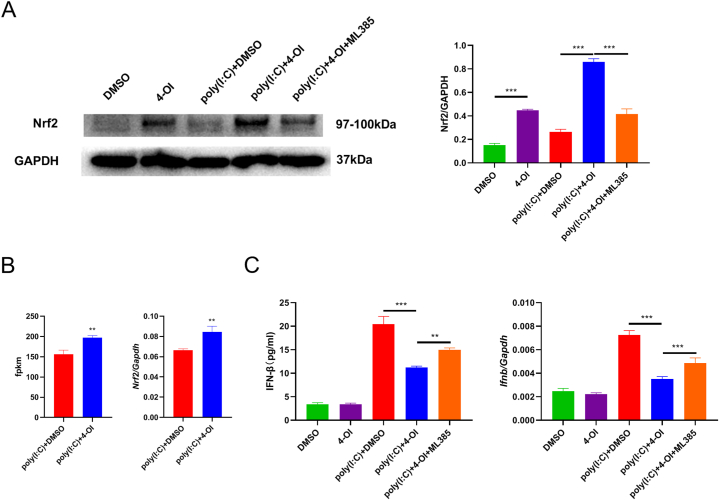
**Nrf2 inhibitor ML385 reversed the inhibitory effect of 4-OI.** A. We used 0.25 mM 4-OI or DMSO to pretreat BMDM for 2 h and then added poly (I:C) (100 μg/mL) for 4 h. The dosage of ML385 in the inhibitor-treatment group was 10 μM. A. The cells were collected to isolate proteins. The expression of Nrf2 was detected by WB. GAPDH was used as a contrast. The relative gray value expressed by Nrf2 was calculated by Image J software. For uncropped images of the membranes, see Supplementary Fig. 1. B. The fpkm value of Nrf2 was sequenced by transcriptome and the expression of Nrf2 was detected by quantitative PCR. C. IFN-β secretion was detected by ELISA, and the mRNA expression of IFN-β was detected by quantitative PCR. The mean values between the two groups were compared by unpaired *t*-test. Analysis of variance was applied for comparison among ≥3 groups, and multiple tests of mean were performed using Tukey's test. **p < 0.01; ***p < 0.001, (n = 3).

As Hmox1 and Gss among the upregulated genes are considered Nrf2-related genes, we investigated the effect of 4-OI on Nrf2 protein expression. After 4-OI was added, the increase in Nrf2 protein levels could be detected with or without the addition of poly(I:C). We treated BMDM with an Nrf2 inhibitor, ML385, and found that Nrf2 protein expression decreased significantly after ML385 addition (Fig. 6A). In transcriptome sequencing, fpkm increased only slightly after 4-OI was added. Similarly, quantitative PCR also revealed that 4-OI addition only slightly upregulated Nrf2 mRNA expression (Fig. 6B), suggesting that 4-OI exerts its effect on macrophage Nrf2 mainly through the regulation of its protein level. ML385 partially reversed the inhibitory effect of 4-OI on poly(I:C)-induced IFN-β secretion and mRNA expression (p < 0.001, Fig. 6C).

### 3.6 siRNA knockdown of Nrf2 partially reverses the inhibitory effect of 4-OI on poly(I:C)-induced IFN-β secretion from BMDM

**Fig. 7:**
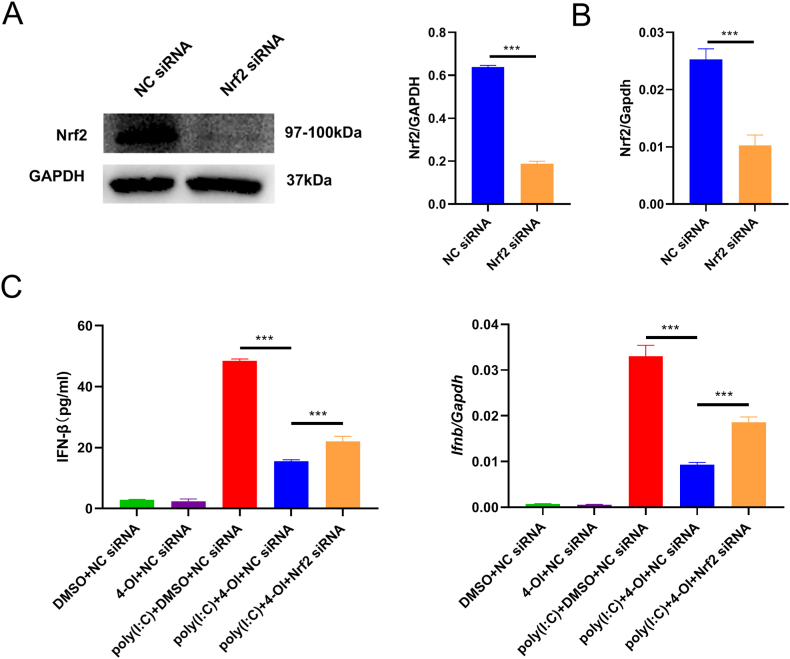
**Inhibitory effect of Nrf2 siRNA on the reversing effects of 4-OI.** BMDM were transfected with NC or Nrf2 siRNA. After 48 h of transfection, BMDM was pretreated with 0.25 mM 4-OI or DMSO for 2 h and then treated with poly(I:C) for 4 h. A. The cells were collected and the expression of Nrf2 was detected by WB, using GAPGH as a contrast. The relative GAPDH gray value expressed by Nrf2 was calculated by Image J. For uncropped images of the membranes, see Supplementary Fig. 2. B. RNA extracted from the cells was collected and reversed into cDNA, followed by the detection of Nrf2 expression by quantitative PCR. C. Quantitative PCR was used to detect the mRNA expression of IFN-β. The supernatant was collected and the IFN-β concentration was detected by ELISA. The mean values between the two groups were compared by unpaired *t*-test. Analysis of variance was applied for comparison among ≥3 groups, and multiple tests of mean were performed using Tukey's test. ***p < 0.001, (n = 3).

We used siRNA to knock down Nrf2 expression. As Nrf2 expression was higher in the presence of poly(I:C) and 4-OI, we used poly(I:C) and 4-OI to treat BMDM and then detected the effect of siRNA-mediated Nrf2 knockdown. The Nrf2 protein was obviously downregulated after siRNA was used (p < 0.001, Fig. 7A). Quantitative PCR also confirmed the effect of Nrf2 knockdown (p < 0.001, Fig. 7B). Then, we found that IFN-β mRNA expression was significantly upregulated after Nrf2 knockdown (p < 0.001, Fig. 7C). Moreover, the ELISA results showed that Nrf2 knockdown reversed the inhibitory effect of 4-OI on IFN-β (p < 0.001, Fig. 7C). Nrf2 knockdown could only partially reverse this effect (Fig. 7C).

## 4 Discussion

We here discovered that 4-OI, a derivative of itaconate, could suppress IFN-β secretion from poly(I:C)-induced mouse BMDM, and Nrf2 was believed to play a critical role in this process. Because IFN-β is the key link in the antiviral reaction, this effect of 4-OI suggests that itaconate may be a key player in the regulation of the host's anti-RNA virus reaction.

Like lipopolysaccharide- and cGAMP-induced macrophages [14,15], poly(I:C)-induced IFN-β can also be inhibited by 4-OI. Because of the key role of IFN-β in the antiviral response, 4-OI might be a potential drug for reducing excessive antiviral response. At present, whether itaconate derivatives can be used in the clinic needs to be explored further. Fumarate is another key metabolite in the TCA cycle [16,17], and its derivative dimethyl fumarate has been used to treat multiple sclerosis [18,19]. Thus, the usage of 4-OI for the treatment of some specific diseases is possible. Nevertheless, itaconate may have the potential risk of inducing ferroptosis [20], although another study suggested that itaconate inhibits ferroptosis [21]. The dosage of 4-OI should be used cautiously in the clinical treatment. On the other hand, these results suggest that itaconate is an inhibitor of TLR3 activation-induced IFN-β expression in vivo and regulates the antiviral response in vivo. Because itaconate expression is generally induced by infection [10,22], it may be the key molecule regulating IFN-β expression through negative feedback in vivo.

In the transcriptome sequencing analysis, the upregulated and downregulated genes were largely different. GO and KEGG enrichment of differently expressed genes revealed that many downregulated genes were related to inflammatory and antivirus effects, whereas many upregulated genes were related to metabolism. Thus, 4-OI seems to have multiple functions, that is, not only affecting IFN-β and inflammation but also regulating the metabolism of various substances. Because itaconate is a key metabolite [[23], [24], [25]], these multifaceted effects are reasonable. This also indicated that the use of itaconate as a target might lead to a series of side effects in the body. The study of its downstream targets will contribute to the use of itaconate in clinics.

4-OI regulates two different functions at the same time, that is, anti-inflammation and antioxidants [26,27]. Notably, many downstream genes of Nrf2 were present among the upregulated genes. Furthermore, 4-OI is considered to activate Nrf2 by affecting Kelch-Like ECH Associated Protein 1 [14]. Therefore, we studied the effect of an Nrf2 inhibitor and siRNA on poly(I:C)-induced IFN-β secretion. Based on the results, Nrf2 played an important role in the inhibitory effect of 4-OI on IFN-β secretion. Nrf2 regulated the secretion of IFN-β induced by dsDNA through the expression of STING [15]. STING recognizes cGAMP downstream of double-stranded DNA; therefore, Nrf2 may regulate IFN-β induced by dsRNA through other pathways. It was reported Nrf2 regulated the secretion of inflammatory factors induced by lipopolysaccharides by binding to the proximity of these inflammatory factors, thereby inhibiting the recruitment of RNA Pol II [28]. Nrf2 might regulate IFN-β in a similar way. Moreover, Nrf2 affected type I interferon secretion by regulating macrophages intermediate metabolism [29], suggesting that the regulation of IFN-β by Nrf2 is closely related to the metabolic process.

ML385 and Nrf2 siRNA partially reversed the inhibitory effect of 4OI on IFN-β, indicating that Nrf2 may only play a partial regulatory role in the mediation effect of 4OI and that there may be other regulators such as succinate dehydrogenase [22], IκBζ-ATF3 [11], GAPDH [30], Tet methylcytosine dioxygenase 2 [31], or Janus kinase 1 [32]. Nevertheless, our study revealed that Nrf2 activation play a part in the regulatory effect of 4OI on poly(I:C)-induced IFN-β secretion.

Through the transcriptome analysis of 4-OI-induced genes, we found an overlap between 4-OI-regulated genes and DI-regulated genes in our former studies, such as Edn1, Layn, and Gss, including both upregulated and downregulated genes [13]. DI-treated macrophages in that study were induced with lipopolysaccharide + adenosine triphosphate [13], and a similar regulation of multiple genes was observed. This indicated that itaconate derivatives have many similar biological functions. Some studies have shown that 4-OI and DI may exhibit some differences, that is, 4-OI is more likely to activate Nrf2, while DI is more closely related to ATF3 [11,14,33]. Despite this, because of their similar molecular structures, considerable consistency is observed in their functions [[34], [35], [36], [37]].

In conclusion, 4-OI could inhibit poly(I:C)-induced IFN-β secretion in mouse BMDM, and Nrf2 played an important role in this inhibitory effect. As both too-strong and too-weak antiviral reactions are unfavorable, itaconate as the key target for regulating antiviral reactions is the focus of follow-up research.

## Ethics statement

The animal procedures were approved by the Institutional Animal Care and Use Committee, Zhejiang Center of Laboratory Animals(ZJCLA-IACUC-20140002).

## Funding statement

This study was supported by 10.13039/501100004731Zhejiang Provincial Natural Science Foundation of China (LY21H150002, LY22H250001), the 10.13039/501100001809National Natural Science Foundation of China (81971325), the 10.13039/501100008856Department of Health of Zhejiang Province (2020KY388).

## Data availability statement

Data associated with this study has been deposited at NCBI Sequence Read Archive under the accession number PRJNA992638.

## CRediT authorship contribution statement

**Ying-Xing Yue:** Data curation, Investigation, Writing – original draft. **Bing-Bing Jia:** Investigation. **Ji-Rong Wang:** Investigation. **Ying-Zheng Weng:** Investigation. **Gen-Xiang Mao:** Data curation. **Yan Lu:** Investigation. **Jing Yan:** Conceptualization, Writing – original draft. **Zhou-Xin Yang:** Conceptualization, Data curation, Investigation, Writing – review & editing.

## Declaration of competing interest

The authors declare no competing interests.
