# Supplementary material for: 4-Octyl itaconate inhibits poly(I:C)-induced interferon-β secretion in mouse bone marrow-derived macrophages partially by activating Nrf2
Source: Heliyon. 2023 Dec 1;9(12):e23001. doi: 10.1016/j.heliyon.2023.e23001 (PMC10703706; doi:10.1016/j.heliyon.2023.e23001)
Supplement: Multimedia component 1 [file mmc1.docx]

**4-Octyl itaconate inhibits poly(I:C)-induced interferon-β secretion in mouse bone marrow-derived macrophages partially by activating Nrf2**

**Supplementary Figures:**

**
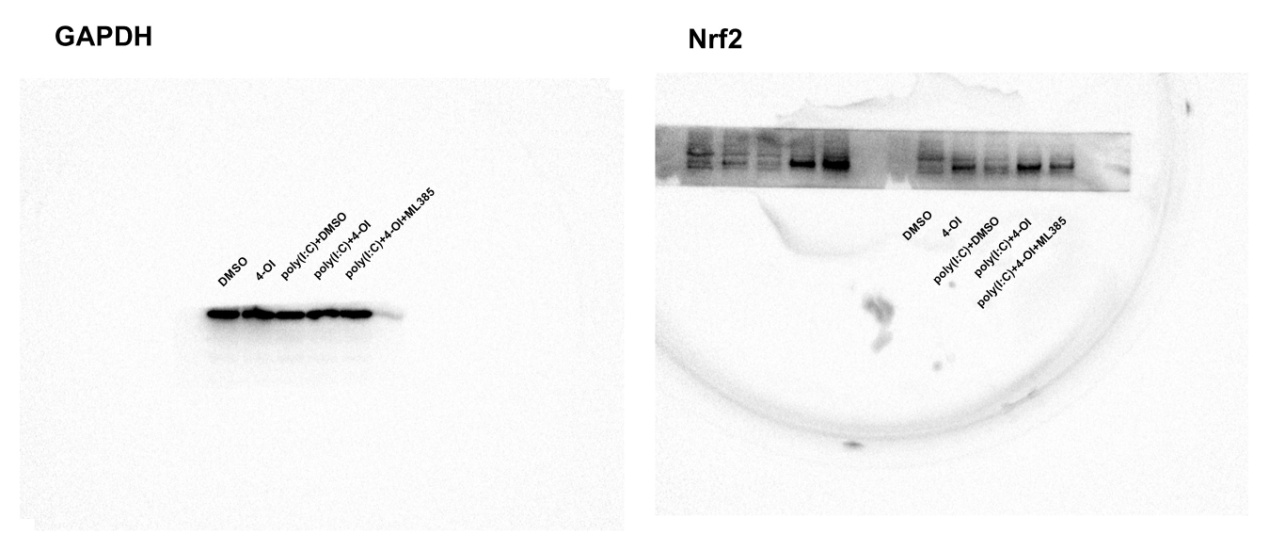
**

**Supplementary Figure 1:** Uncropped images of the membranes for Figure 6A


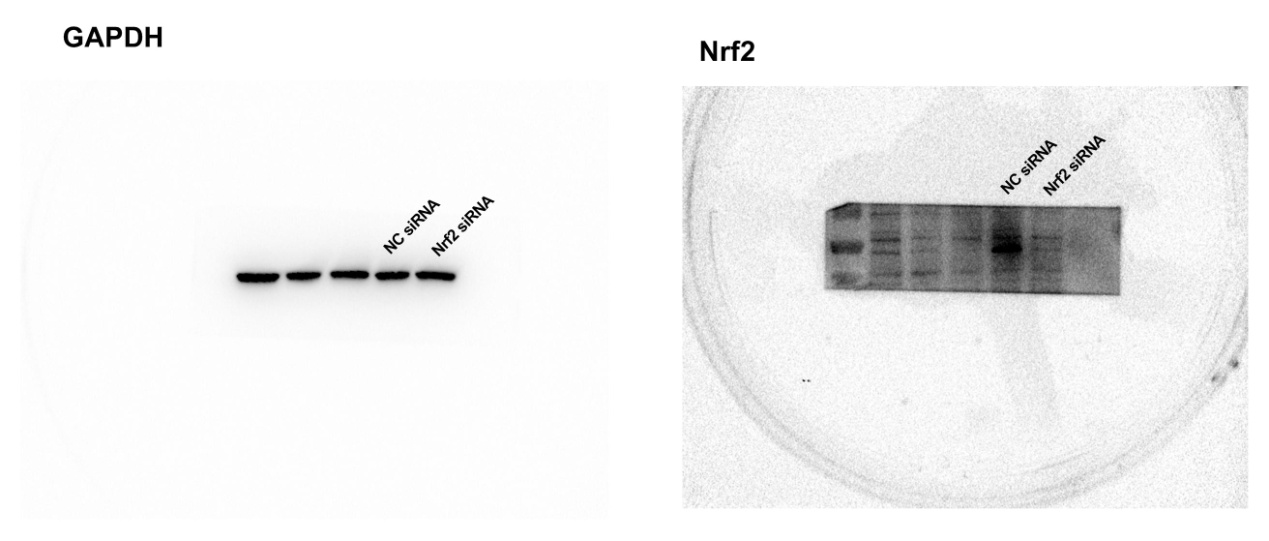


**Supplementary Figure 2:** Uncropped images of the membranes for Figure 7A
